# Changes in leukocytes and CRP in different stages of major depression

**DOI:** 10.1186/s12974-022-02429-7

**Published:** 2022-04-04

**Authors:** Deepti Singh, Paul C. Guest, Henrik Dobrowolny, Veronika Vasilevska, Gabriela Meyer-Lotz, Hans-Gert Bernstein, Katrin Borucki, Alexandra Neyazi, Bernhard Bogerts, Roland Jacobs, Johann Steiner

**Affiliations:** 1 Department of Psychiatry and Psychotherapy, Otto-von-Guericke-University Magdeburg, Leipziger Str. 44, 39120 Magdeburg, Germany; 2 Laboratory of Translational Psychiatry, Otto-von-Guericke-University Magdeburg, Magdeburg, Germany; 3 Laboratory of Neuroproteomics, Department of Biochemistry and Tissue Biology, University of Campinas (UNICAMP), Campinas, Brazil; 4 Institute of Clinical Chemistry and Pathobiochemistry, Otto-von-Guericke-University Magdeburg, Magdeburg, Germany; 5 Department of Psychiatry, Social Psychiatry and Psychotherapy, Hannover Medical School, Hannover, Germany; 6 Center for Behavioral Brain Sciences (CBBS), Magdeburg, Germany; 7 Salus Institute, Magdeburg, Germany; 8 Department of Rheumatology and Clinical Immunology, Hannover Medical School (MHH), Hannover, Germany; 9 Center for Health und Medical Prevention (CHaMP), Magdeburg, Germany; 10 German Center for Mental Health (DZP), Center for Intervention and Research on Adaptive and Maladaptive Brain Circuits Underlying Mental Health (C-I-R-C), Jena-Magdeburg-Halle, Germany

**Keywords:** Neutrophils, Neutrophil–lymphocyte ratio (NLR), Monocytes, Monocyte–lymphocyte ratio (MLR), Eosinophils, Innate immunity, Adaptive immunity

## Abstract

**Background:**

We recently reported increased levels of neutrophils, monocytes and C-reactive protein (CRP) correlated with symptom severity in acute schizophrenia. Here, we investigated if a similar pattern of innate immune system activation occurs in major depression (MD).

**Methods:**

We assessed differential blood counts, CRP, depression symptoms (HAMD-21) and psychosocial functioning (GAF) in controls (*n* = 129) and patients with first (FEMD: *n* = 82) or recurrent (RMD: *n* = 47) disease episodes of MD at baseline (T0; hospital admission) and after 6-weeks treatment (T6).

**Results:**

Considering smoking, BMI and gender as covariates, neutrophils (FEMD: *p* = 0.034, RMD: *p* = 0.034) and CRP (FEMD: *p* < 0.001, RMD: *p* = 0.021) were higher, and eosinophils (FEMD: *p* = 0.005, RMD: *p* = 0.004) lower in patients versus controls at T0. Baseline lymphocyte counts were elevated in RMD (*p* = 0.003) but not FEMD. Results were confirmed by analyses of nonsmokers. At follow-up, eosinophils rose significantly in FEMD (*p* = 0.011) but no significant changes were observed in RMD. Improvement in HAMD-21 correlated with T0–T6 changes of neutrophil counts in FEMD (*r* = 0.364, *p* = 0.024). Compared with our previous schizophrenia study, raised baseline neutrophil and reduced eosinophil counts in MD had smaller effect sizes and treatment had a weaker association with T0-T6 changes in neutrophils. In addition, lymphocytes were elevated at T0 in recurrent MD but not in schizophrenia patients.

**Conclusions:**

These findings suggest that innate immunity may be involved in early stages of MD, and adaptive immunity may be involved in chronic disease. Thus, further studies may lead to new disease stage-dependent MD treatment strategies targeting different aspects of immune system activation.

**Supplementary Information:**

The online version contains supplementary material available at 10.1186/s12974-022-02429-7.

## Introduction

Major depression (MD) shares clinical similarities with cytokine-induced sickness behavior, which is a set of adaptive behavioral and mental changes that occur in response to an infection. In this scenario, immune cells produce proinflammatory cytokines, such as interleukin (IL)-1β, IL-6, interferon-gamma (IFN-γ) and tumor necrosis factor alpha (TNF-α) that can act on the brain and cause depressed mood, emotional lability, poor concentration, loss of drive or motivation, social withdrawal, lack of appetite, sleep disturbances, and decreased personal hygiene [1]. Meta-analyses have shown that IL-1β, IL-6 and TNF-α are likewise increased in the peripheral blood of psychiatric patients with a current episode of MD [2–4]. Shared proinflammatory cytokine pathways explain the partially overlapping clinical phenomenology of sickness behavior and MD, as the presence of proinflammatory cytokines in the brain can lead to decreased availability of serotonin and its precursor tryptophan [5]. However, the biological similarity between cytokine-induced sickness behavior and MD is only partial. The former is an adaptive response to infection that is reversible but this is not the case with MD. Thus, it has been proposed that MD represents a maladaptive version of cytokine-induced sickness [6].

In support of an involvement of inflammatory triggers in depression, large epidemiological studies have identified previous severe infections or autoimmune diseases as a risk factor for mood disorders [7, 8]. This appears to occur through direct effects of the infections or inflammation on the brain, and via alterations of the microbiome or other environmental factors. During acute infections, the innate immune system is activated as the first line of defense. With regard to cellular components, most studies of MD have analyzed the mononuclear phagocyte system (MPS) and a key role for activated microglia and circulating monocytes has been postulated [9]. A few studies have been published on neutrophil–lymphocyte ratios in mood disorders [10], but generally less is known about the involvement of granulocytes in MD, which represent the dominant cellular component of the innate immune system. This may be due to a stronger focus of psychoimmunology on peripheral blood mononuclear cells (PBMCs) [11]. Granulocytes (neutrophils, basophils, and eosinophils) account for approximately 50–80% of all leukocytes, but were often discarded and not analyzed after isolation of PBMCs in previous studies.

Recently, we analyzed white blood cell counts (WBCs) along with neutrophil and monocyte–macrophage activation markers in individuals with acute first-episode and relapsed schizophrenia compared to controls [12]. Neutrophil counts were increased with large effect sizes and monocyte counts were raised with small-to-medium effect sizes during acute illness. These changes were accompanied by higher levels of four neutrophil and monocyte activation markers in serum, including neutrophil gelatinase associated lipocalin (NGAL) and macrophage inflammatory protein-1 alpha (MIP-1α, synonym CCL3) which are associated with bacterial infections. In parallel, eosinophil counts were slightly reduced. These findings imply an innate immune system activation during psychosis and partially normalized after 6 weeks of treatment. Neutrophil counts correlated with the severity of positive symptoms, suggesting a pathophysiological importance of the innate immune system in the context of psychosis.

For the present study, we investigated whether leukocyte and C-reactive protein (CRP) changes occur in different stages of MD. We included patients with first-episode MD (FEMD) and recurrent MD (RMD) and carried out baseline and follow-up assessments after a 6-week treatment period. To exclude medication effects as potential confounders, we focused on FEMD patients who were drug-naïve and RMD patients who had not been medicated for at least 6 weeks prior to baseline assessment. The aims were to determine if: (1) WBCs in patients with a current disease episode of MD are altered in a similar way to schizophrenia; (2) WBCs change after 6-weeks treatment; and (3) WBCs correlate with psychopathological assessments, cumulative drug dosage and type of antidepressant medication. In parallel, we also (4) controlled for age, gender, BMI, tobacco smoking, disease duration and stress (basal cortisol blood levels) as potential confounders. Finally, following two studies by Lamers et al. [13, 14], we (5) examined whether patients who fulfill DSM-IV-TR criteria for atypical MD differed from other MD patients in terms of distribution of WBCs in the acutely ill unmedicated state [15].

## Materials and methods

### Samples

Specimens came from the scientific blood bank of the Department of Psychiatry and Psychotherapy, Otto-von-Guericke-University Magdeburg, Germany [12, 16–18] and were collected from sequentially-admitted acutely-ill inpatients with MD (March 2008 to January 2020; *n* = 129). FEMD participants (*n* = 82) were drug-naïve at baseline (T0) and RMD participants (*n* = 47) were without any psychiatric medication for at least 6 weeks. Controls (*n* = 129) were healthy blood donors, hospital staff and their relatives, and came from the same collection period (Table 1).

**Table 1:** Demographic and clinical parameters (plus available cortisol data)

Variables	FEMD	RMD	Controls	Test	Test value	*p*-value	*p*-FEMD/C	*p*-RMD/C	*p*-RMD/FEMD
Age (years)	33.5 (25.0;46.3;82)	42.0 (30.0;52.0;47)	38.0 (28.0;47.5;129)	*H*-test	KW = 5.15	0.076	0.214	0.214	0.082
Disease duration (years)	0 (0;0;82)	4.5 (2.0;9.8;32)	–	*U*-test	*W* = 82.0	**< 0.001**			
Gender (female/male)	43/39	33/14	77/52	Chi-Square	*χ*^2^ = 3.93	0.140	0.371	0.371	0.221
BMI_T0	23.1 (20.3;25.6;82)	25.0 (21.4;27.6;47)	24.1 (21.8;27.2;129)	*H*-test	KW = 6.40	**0.041**	0.054	0.431	0.054
BMI_T6	24.0 (21.3;27.3;63)	24.8 (22.3;27.8;36)	–	*U*-test	*W* = 997.5	0.323			
Test value	*W* = 494	*W* = 149							
T0–T6 *p*-value	**0.008**	0.088							
Smoking status (yes/no)	42/40	22/24	29/100	Chi-Square	*χ*^2^ = 21.22	**< 0.001**	**< 0.001**	**0.003**	0.854
Cigarettes/day-T0	4.5 (0;20.0;82)	0 (0;15;46)	0 (0;0;129)	*H*-test	KW = 29.61	**< 0.001**	**< 0.001**	**< 0.001**	0.337
Cigarettes/day-T6	0 (0;15.0;63)	0 (0;14.3;36)	–	*U*-test	*W* = 1219.0	0.506			
Test value	*W* = 109	*W* = 36.5							
T0–T6 *p*-value	0.129	0.788							
AMI-T6 (g)	4.10 (2.81;5.69;54)	3.60 (2.69;6.44;31)	–	*U*-test	*W* = 801.0	0.853			
HAMD-21-T0	19.0 (14.8;23.0;82)	22.0 (15.0;29.0;47)	–	*U*-test	*W* = 1637.0	0.156			
HAMD-21-T6	6.0 (3.0;10.0;63)	8.0 (6.0;13.0;37)	–	*U*-test	*W* = 760.5	**0.004**			
Test value	*W* = 1759	*W* = 660							
T0–T6 *p*-value	**< 0.001**	**< 0.001**							
GAF-T0	50.0 (48.5;60.0;80)	50.0 (41.0;60.0;47)	–	*U*-test	*W* = 2042.0	0.415			
GAF-T6	70.0 (65.8;80.8;60)	65.0 (60.0;76.5;37)	–	*U*-test	*W* = 1398.0	**0.032**			
Test value	*W* = 76.5	*W* = 35							
T0–T6 *p*-value	**< 0.001**	**< 0.001**							
Cortisol-T0 (ng/mL)	330 (181;713;37)	343 (166;1130;20)	229 (171;853;46)	*H*-test	KW = 0.665	0.717	0.823	0.823	0.874
Cortisol-T6 (ng/mL)	212 (140;532;27)	218 (145;818;13)	–	*U*-test	*W* = 149.5	0.461			
Test value	*W* = 318	*W* = 55							
T0–T6 *p*-value	**0.001**	0.542							

Data are presented as median (quartile 1; quartile 3; sample size) or number of cases

*AMI* cumulative amitriptyline dosage, *HAMD* 21-item Hamilton Scale for Depression, *GAF* Global Assessment of Functioning

Significant *p*-values are highlighted in bold font

Exclusion criteria were alcohol or substance abuse, depression induced by other medical conditions, history of immune disease or immunotherapy [17]. Controls were screened for personal or family history of neuropsychiatric disorders using the Mini-International Neuropsychiatric Interview (MINI) and excluded in case of disease [19]. Procedures were approved by the local institutional review board and written informed consent was obtained.

Blood analyses and psychopathological assessments (21-item Hamilton depression scale [HAMD-21], Global Assessment of Functioning [GAF]) were performed at admission (baseline). Follow-up assessments after 6 weeks (T6) were available for 100 patients. The types and cumulative dosages of antidepressant drugs taken from baseline to follow-up were documented and converted into amitriptyline (AMI) equivalents [20–22]. Eighty-four subjects (FEMD *n* = 53, RMD *n* = 31) were medicated for 6 weeks after baseline assessment (Additional file 1: Table S1). Sixteen patients were treated with psychotherapy and received no antidepressant drugs (FEMD *n* = 10, RMD *n* = 6). Assessment of which patients suffered from atypical depression at T0 was carried out using DSM-IV-TR criteria [15].

Blood samples were obtained from fasting subjects at 08:00 and collected into BD Vacutainer™ tubes (Becton Dickinson; Heidelberg, Germany). EDTA blood tubes were used to determine the WBC counts within 1 h. Serum tubes were allowed to clot for 2 h and then centrifuged at 1000×*g* for 10 min. The supernatants were collected, aliquoted and stored at − 80 °C.

### Blood analyses

WBCs were determined using an XN-3000 automated counter (Sysmex Corporation; Kobe, Japan). Serum CRP concentrations were quantified by a Cobas 8000 c701 modular analyzer (Roche Diagnostics; Basel, Switzerland). For 103 subjects (FEMD: *n* = 37, RMD: *n* = 20, controls: *n* = 46), basal serum cortisol measurements were available from previous studies (Table 1) [23, 24]. These had been quantified using the Immulite 2000 system (Siemens Healthcare Diagnostics; Eschborn, Germany), which is a fully automatic random access chemiluminescence-enhanced enzyme immunoassay protocol. Intra- and inter-assay coefficients of variation were < 5% for all assays.

### Statistics

Data analyses were carried out using the statistical software packages R (v4.0.5, http://www.r-project.org) and SPSS Statistics (v26; Armonk, NY, USA). Chi-square tests were used to calculate group differences regarding gender and smoking status (yes/no). Most data were not normally distributed as indicated by Shapiro–Wilk tests. Thus, differences between groups were calculated by non-parametric Kruskal–Wallis *H*-tests and Mann–Whitney *U*-tests.

WBC counts (i.e., neutrophil, eosinophil, basophil, monocyte, lymphocyte counts) and CRP were the 6 *main outcome parameters*. Supplementary, two ratios were calculated, the neutrophil:lymphocyte ratio (NLR) and monocyte:lymphocyte ratio (MLR) to allow comparisons with other publications. Correlations of HAMD-21, GAF and cortisol with the main outcome parameters were assessed by *Spearman rank tests*.

The Benjamini–Hochberg method was used to control the False Discovery Rate (FDR) for multiple hypothesis testing. We controlled the test for differences between the 3 diagnostic groups at T0 for 6 repeated rounds of comparison (main outcome parameters) and for 2 repeated comparisons in the case of ratios (NLR and MLR) [25]. Post-hoc comparisons between 2 groups at T0 aimed at identifying differences between pairs of diagnoses and were controlled for 3 rounds of comparison. Changes over time T0–T6 were corrected for 6 (main outcome parameters) and 2 (NLR and MLR) repeated rounds of comparisons, respectively. Spearman correlation matrices were FDR-corrected for the number of correlations calculated.

Factors such as age, gender, BMI, smoking (number of cigarettes/day), disease duration and cortisol blood levels may interfere with the diagnostic group differences in WBC counts or CRP. An overcorrected statistical model including all these factors as covariates could lead to artifacts [26]. Therefore, *probatory analyses of covariance (ANCOVA) using an Aligned Rank Transformation (ART)* with testwise inclusion of age/gender/BMI/smoking/disease duration/cortisol as single covariates were performed (Table 2). The identified relevant confounding factors (significant as a single covariate in Table 2) for the calculation of diagnosis-dependent differences in WBC counts and CRP were included as covariates in the *final ART model*. Smoking habits of the disease groups did not change significantly over time and repeated measures ART analyses were used to determine changes from T0 to T6. Further validation against interference of our results by smoking was performed by calculating all diagnosis-related differences separately for the non-smoking participants only.

**Table 2:** Covariate-effect analysis by probatory ART-analyses

Variables	Effect of single covariates in probatory ART-analyses (*p*-values)						
	Age	Gender	BMI	Smoking (cigarettes/day)	Disease duration	Cortisol	Relevant covariate(s) for final ART-analyses
Neutrophils-T0	0.644	0.872	0.157	**< 0.001**	0.539	0.499	Smoking
Neutrophils-T6	0.898	0.855	0.368	**0.010**	0.928	0.990	Smoking
Eosinophils-T0	0.644	0.072	**0.014**	**< 0.001**	0.590	0.499	BMI + smoking
Eosinophils-T6	0.896	0.268	0.330	**0.023**	0.772	0.723	Smoking
Basophils-T0	0.644	0.765	0.532	0.130	0.515	0.866	nrci
Basophils-T6	0.178	0.268	0.368	0.170	0.833	0.875	nrci
Monocytes-T0	0.644	**0.009**	**0.035**	**0.002**	0.539	0.892	Gender + BMI + smoking
Monocytes-T6	0.896	0.082	0.395	**0.010**	0.626	0.723	Smoking
Lymphocytes-T0	0.363	0.552	**0.003**	**0.001**	0.515	0.892	BMI + smoking
Lymphocytes-T6	0.896	0.855	0.213	**0.023**	0.772	0.723	Smoking
CRP-T0	0.999	0.062	**< 0.001**	0.094	0.662	0.776	BMI
CRP-T6	0.896	0.906	**< 0.001**	0.226	0.626	0.723	BMI

Factors that had a significant influence on respective cell counts or CRP were included as covariates in the final ART model

*Nrci* no relevant covariate identified

Significant *p* values are highlighted in bold font

Cohen’s *d* was used to assess effect size (ES) of the ART-analyses. *d* ≥ 0.2, ≥ 0.5 and ≥ 0.8 were considered as small, medium and large ES, respectively [27]. All statistical tests were two-tailed with *p* < 0.05 considered significant and *p* < 0.10 as trending towards significance.

## Results

### Demographic data

As expected, FEMD patients tended to be younger (*p* = 0.082) and had a significantly shorter disease duration (*p* < 0.001) than RMD patients (Table 1). Overall, there was a slight difference in BMI between groups (*p* = 0.041). Both patient cohorts contained more tobacco smokers than controls (FEMD vs. controls: *p* < 0.001; RMD vs. controls: *p* = 0.003). No significant differences were observed regarding gender distribution.

### Identification of relevant confounding parameters

As summarized in Table 2, according to probatory ART-analyses, smoking and BMI were identified as main confounding factors for calculations of diagnosis-dependent differences in WBC counts and CRP. Smoking affected neutrophil, eosinophil, monocyte and lymphocyte counts while BMI interfered significantly with eosinophil, monocyte, lymphocyte counts and CRP. Gender only affected monocyte counts. Therefore, *smoking, BMI and gender were used as covariates in respective final ART-analyses*. Age, disease duration and cortisol blood levels had no significant effect in our sample.

### Baseline/T0

No significant differences were observed regarding severity of illness in terms of HAMD-21 and GAF scores at baseline.

**Fig. 1:**
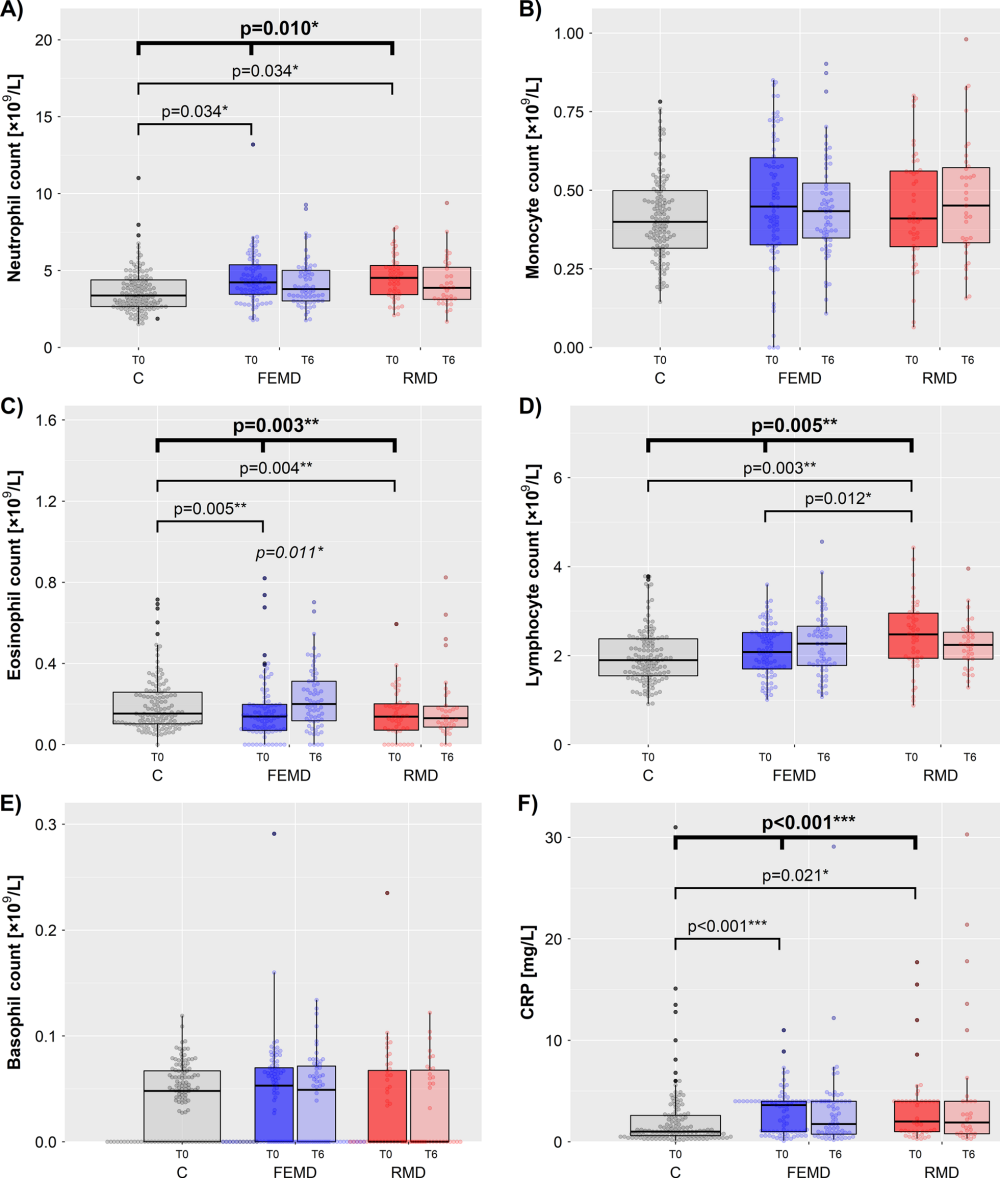
**A** Neutrophil, **B** eosinophil, **C** basophil, **D** monocyte and **E** lymphocyte counts (×10^9^/L), and **F** CRP levels (mg/L) in FEMD, RMD and controls. *p < 0.05, **p < 0.01, ***p < 0.01

Final ART-analyses revealed significant differences between the three diagnostic groups regarding neutrophil (*p* = 0.010), eosinophil (*p* = 0.003), lymphocyte (*p* = 0.005) and CRP measures (*p* < 0.001) at baseline (Table 3, Fig. 1). No significant diagnosis-related differences were observed regarding basophil and monocyte counts as well as the NLR and MLR at T0.

**Table 3:** Leukocyte counts and CRP levels

Variables	FEMD	RMD	Control	Final ART-analysis^a^ *p*-value FEMD/RMD/C	Post-hoc ART-analysis^a^ *p*-FEMD/C	Post-hoc ART-analysis^a^ *p*-RMD/C	Post-hoc ART-analysis^a^ *p*-RMD/FEMD
Neutrophils-T0^b^	4.22 (3.43;5.37;81)	4.52 (3.42;5.39;46)	3.37 (2.66;4.40;128)	**0.010**	**0.034**	**0.034**	0.374
Neutrophils-T6^b^	3.79 (3.02;5.06;62)	3.87 (3.12;5.31;35)	–	0.803			
T0–T6 *p*-value	0.104	0.134					
Eosinophils-T0^b^	0.14 (0.07;0.20;81)	0.14 (0.07;0.20;44)	0.15 (0.10;0.26;128)	**0.003**	**0.005**	**0.004**	0.679
Eosinophils-T6^b^	0.20 (0.12;0.32;62)	0.13 (0.08;0.20;34)	–	0.676			
T0–T6 *p*-value	0.011*	0.572					
Basophils-T0^b^	0.05 (0.00;0.07;81)	0 (0;0.07;44)	0.05 (0;0.07;128)	0.445	0.518	0.470	0.470
Basophils-T6^b^	0.05 (0.00;0.07;62)	0 (0;0.07;34)	–	0.676			
T0–T6 *p*-value	0.408	0.975					
Monocytes-T0^b^	0.45 (0.32;0.61;78)	0.41 (0.32;0.56;44)	0.40 (0.32;0.50;128)	0.519	0.560	0.732	0.732
Monocytes-T6^b^	0.43 (0.34;0.54;60)	0.45 (0.33;0.58;35)	–	0.676			
T0–T6 *p*-value	0.298	0.720					
Lymphocytes-T0^b^	2.08 (1.66;2.52;81)	2.48 (1.93;2.96;46)	1.90 (1.52;2.38;128)	**0.005**	0.492	**0.003**	**0.012**
Lymphocytes-T6^b^	2.27 (1.77;2.69;62)	2.24 (1.92;2.55;35)	–	0.785			
T0–T6 *p*-value	0.104	0.171					
CRP-T0 (mg/L)	3.61 (1.00;4.00;82)	2.00 (1.00;4.00;45)	1.00 (0.60;2.65;129)	**< 0.001**	**< 0.001**	**0.021**	0.260
CRP-T6 (mg/L)	1.75 (0.70;4.00;62)	1.90 (0.80;4.00;32)	–	0.676			
T0–T6 *p*-value	0.199	0.975					
Neutrophil/lymphocyte-T0	2.00 (1.53;2.77;81)	1.67 (1.38;2.32;46)	1.80 (1.37;2.25;128)	0.164	0.129	0.637	0.129
Neutrophil/lymphocyte-T6	1.78 (1.28;2.62;62)	1.76 (1.46;2.20;35)	–	0.929			
T0–T6 *p*-value	**0.032**	0.835					
Monocyte/lymphocyte-T0	0.23 (0.15;0.32;78)	0.20 (0.14;0.25;44)	0.21 (0.16;0.28;128)	0.164	0.442	0.442	0.418
Monocyte/lymphocyte-T6	0.20 (0.15;0.26;60)	0.23 (0.15;0.28;35)	–	0.685			
T0–T6 *p*-value	**0.032**	0.146					

Complementary neutrophil:lymphocyte and monocyte:lymphocyte ratios are displayed in the lower part of this table (for interested readers)

Data are presented as median (quartile 1; quartile 3; sample size)

*CRP* C-reactive protein

Significant *p*-values are highlighted in bold font

^a^Final ART-analyses with relevant covariates smoking, BMI, gender (from Table 2)

^b^×10^9^/L

Post-hoc two-group ART-analyses showed significantly higher neutrophil counts in FEMD (*p* = 0.034, *d* = 0.329) and RMD (*p* = 0.034, *d* = 0.373) compared to controls with small effect sizes, and no difference was observed between the two psychiatric groups (Table 3, Fig. 1). Eosinophil counts were lower in FEMD (*p* = 0.005, *d* = − 0.166) and RMD (*p* = 0.004, *d* = − 0.211). Lymphocyte counts were significantly higher in RMD vs. FEMD patients (*p* = 0.012, *d* = 0.278) and vs. controls (*p* = 0.003, *d* = 0.386) with small effect sizes but no significant difference was observed between FEMD and controls. No significant differences were observed in basophil and monocyte counts. CRP levels were elevated in both patient groups compared to controls (FEMD vs controls: *p* < 0.001, *d* = 0.317, RMD vs. controls: *p* = 0.021, *d* = 0.274), with no significant difference observed between FEMD and RMD.

Supplementary analyses showed no diagnosis-related differences regarding NLR and MLR (Table 3). Moreover, WBCs at T0 did not differ between atypical and typical MD (Additional file 1: Table S2).

### Follow-up/T6

The 6-week treatment period led to a significant improvement of HAMD-21 and GAF scores in FEMD and RMD patients (each *p* < 0.001; Table 1). Symptom severity was significantly lower in FEMD than in RMD patients at T6 (HAMD-21: *p* = 0.004, GAF: *p* = 0.032), although the administered types (χ^2^ = 30, p = 0.224) and cumulative dosages of antidepressant drugs (*p* = 0.853, Table 1) were not significantly different.

A shift towards lower NLR (*p* = 0.032) and MLR (*p* = 0.032) from T0 to T6 was observed in FEMD with a non-significant reduction in neutrophils (*p* = 0.104) and non-significant increase in lymphocytes (*p* = 0.104); Table 3, Fig. 1). Eosinophil counts rose significantly in FEMD patients from T0 to T6 (*p* = 0.011). No significant changes over time were observed in RMD or regarding basophil, monocyte or CRP measures.

### Association of depressive symptoms and GAF with peripheral blood cells and CRP

#### Baseline/T0

Spearman correlation analysis revealed no significant association of HAMD-21 and GAF scores of depressed individuals with granulocyte, monocyte, lymphocyte and CRP measures at baseline.

#### Follow-up/T6

**Fig. 2:**
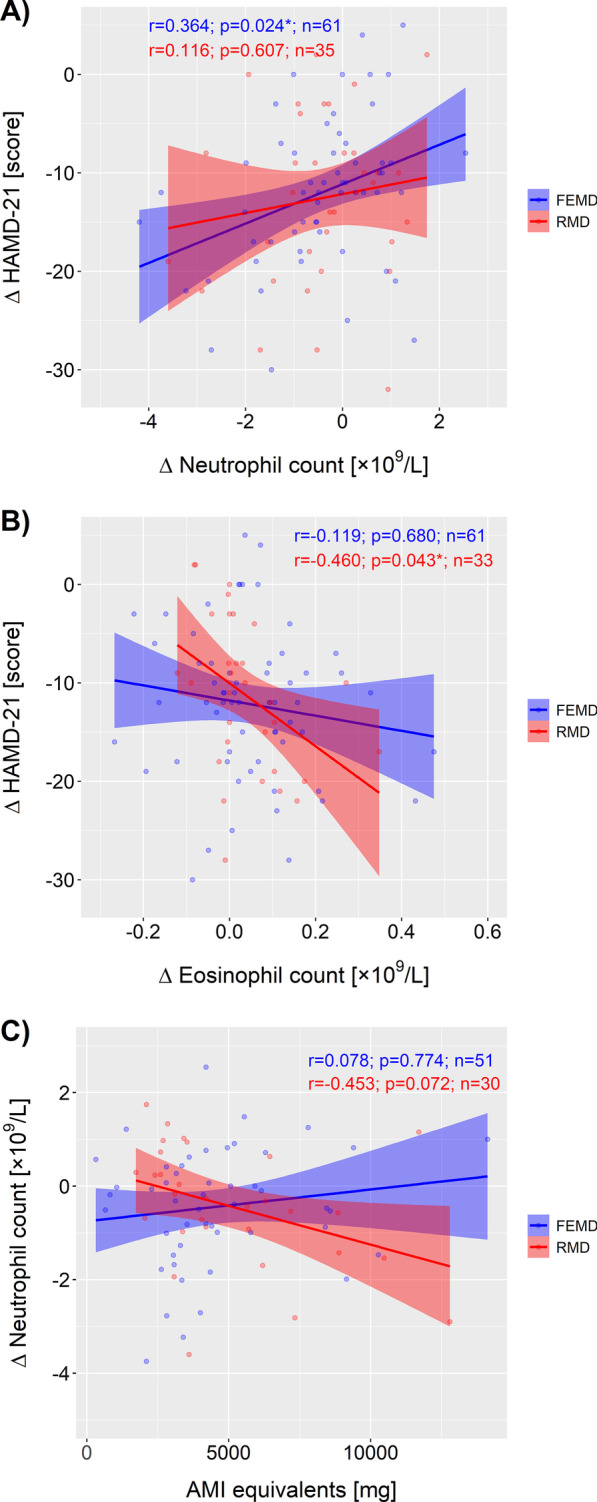
Spearman correlation analyses showing **A** larger treatment-related decreases in HAMD scores (ΔHAMD-21) associated with larger decreases in neutrophils (Δ neutrophil count) and vice versa in FEMD patients only, **B** larger treatment-related decreases in HAMD scores (ΔHAMD-21) associated with larger increases in eosinophils (Δ eosinophil count) and vice versa in RMD participants only. **C** Cumulative amitriptyline (AMI) equivalent dosage correlated at trend level with the reduction in neutrophil count (Δ neutrophil count) after 6 weeks of treatment in RMD patients only. Δ calculations were performed by subtracting T0 from T6 measures, *p < 0.05

Larger treatment-related decreases in HAMD scores were associated with larger decreases in neutrophils and vice versa—in FEMD participants only (*r* = 0.364, *p* = 0.024; Fig. 2a). Conversely, larger treatment-related decreases in HAMD scores were associated with larger increases in eosinophils and vice versa—in RMD participants only (*r* = − 0.460, *p* = 0.043; Fig. 2b). There were no further associations between ΔHAMD-21 or ΔGAF scores and blood cell counts or CRP levels (Additional file 1: Table S3).

### Antidepressant drug association with ΔWBC and ΔCRP measures

Although there were no differences in the distribution of administered medication types or cumulative dosages between FEMD and RMD (see above: Follow-up/T6), cumulative AMI dosage correlated at trend level with the reduction in neutrophil counts in RMD (*r* = 0.453, *p* = 0.072) but not in FEMD (*r* = − 0.078, *p* = 0.774) patients (Fig. 2c). This was not attributable to a specific antidepressant drug type, as shown by separate correlation analyses and no other correlations were found.

### Additional analyses on the possible influence of smoking and stress

A separate analysis focusing only on non-smoking patients and controls confirmed the above diagnostic group differences regarding raised neutrophil and CRP measures and reduced eosinophil counts in both psychiatric groups at baseline (Additional file 1: Table S4). Although not significant, median lymphocyte counts were slightly higher in non-smoking RMD patients compared with controls at T0.

Non-significant higher median cortisol levels were observed in both patient groups compared to controls at baseline (Table 1). A FDR-corrected Spearman correlation matrix between cortisol and WBC counts or clinical scores showed only a correlation of cortisol with eosinophil counts in FEMD patients (*r* = 0.492, *p* = 0.030; Additional file 1: Table S5).

## Discussion

Despite the fact that numerous studies have demonstrated activation of the innate immune system in MD (Additional file 1: Table S6), only one investigated this process at different stages of disease [28]. Consistent with the latter study, which investigated immune responses in FEMD and RMD elderly patients, we observed elevated neutrophil counts in both first-episode and recurrent MD patients compared to controls at baseline. However, our study extended these findings by investigating non-elderly patients who were medication-free at the time of study and by ruling out potential effects of confounding factors such as cigarette smoking and stress. It should be mentioned that two out of the 11 studies highlighted in Additional file 1: Table S6 investigated patients with a current episode of MD [29, 30], one accounted for effects of smoking [31], two excluded smokers [32, 33] and three accounted for stress [31, 34, 35]. In addition, three of these investigations included patients who were antidepressant-free at the time of the study [31, 32, 36]. Nonetheless, this is the first study which has attempted to account for all of these effects.

Our baseline cross-sectional study of MD patients showed similarity with our previous investigation of schizophrenia patients [12] as neutrophil counts were also found to be elevated in both FEMD and RMD (see Additional file 1: Table S7). However, the effect sizes in the current study were small and treatment with antidepressants had a weaker association with partially decreased neutrophil counts compared to the antipsychotic treatment. Nevertheless, these findings suggest a role of neutrophils in depression in line with the finding that transmigration of neutrophils into the brain induces depressive-like behavior in mice [37]. In line with publications on depression in the context of (prolonged) sickness behaviour [1], a study in mice treated with low-dose lipopolysaccharide (LPS) showed that transmigrating neutrophils interacted with activated microglial cells [38]. Furthermore, neutrophils not only seemed to actively interact with microglial processes but also exhibited reverse transendothelial migration back to the bloodstream. These results fit the hypothesis that transient blood–brain barrier (BBB) impairment may play a role during disease episodes of affective and psychotic disorders, at least in patient subgroups [39]. Triggered by neutrophils interacting with elevated CRP, tight junctions can become leaky [40], facilitating proinflammatory cytokines, antineuronal autoantibodies, or peripheral blood cells to cross the BBB and act on brain function. Consequently, disturbances in serotonergic, glutamatergic, noradrenergic, and dopaminergic, neurotransmission can be driven by proinflammatory cytokines, either directly or by modulation of the kynurenine pathway of tryptophan metabolism [39, 41].

Infiltration of neutrophils has been reported in infectious, non-infectious and autoimmune disorders such as rheumatoid arthritis [42], ulcerative colitis [43] and asthma [44]. Neutrophilia in peripheral blood can occur via including increased proliferation and mobilization from bone marrow or vessel walls (marginated cells). The latter effect occurs as an acute response to noradrenalin and cortisol [45]. At baseline, depressed patients tended to have high cortisol levels compared to controls and treatment led to significantly reduced cortisol levels and eosinophil counts in FEMD but not in RMD patients. Moreover, a delay in spontaneous neutrophil death is induced by tobacco smoke and nicotine [46, 47]. Therefore, we considered cortisol levels and cigarette smoking as potential confounders and our finding of an MD-associated increase in neutrophil counts was confirmed.

In addition, we found slightly lower eosinophil counts in both first onset and in MD patients with recurrent disease episodes, similar to our previous findings in schizophrenia [12] (Additional file 1: Table S7). This could be caused by higher stress levels [48]. Previous studies have shown that cortisol induces opposite effects on survival of eosinophils and neutrophils by inducing apoptosis in the former and inhibiting this process in the latter [49–51]. Although meta-analyses have confirmed the association of depression with hypothalamic–pituitary–adrenal (HPA)-axis stress activation and inflammation, there is considerable variability in effect sizes across studies [52–54]. When considering cortisol as a covariate (data not shown), the observed MD-related changes regarding eosinophil counts were confirmed, which suggests that other mechanisms are involved. Notably, the pattern of increased neutrophil along with reduced eosinophil blood counts in MD resembles that of bacterial infections that has been known for many decades as a shift in production toward an increased ratio of neutrophils to eosinophils [55]. Apart from an increased mobilization and generation of neutrophils, an endotoxin-induced reduction of mature eosinophils in the (bone marrow and) blood seems to plays a role here [56]. As bacterial inflammation regresses, there is an increase in eosinophils, historically referred to as “eosinophil-driven dawn of recovery” [57]. Here, a rise in eosinophils was detected at follow-up in FEMD during clinical improvement. Unfortunately, the significance of these findings remains unclear due to lack of data on microbial exposure in this cohort.

In contrast with previous findings regarding different levels of proinflammatory cytokines such as IL-6 and TNF-α in atypical versus other types of MD [13, 14], no differences were observed in the present study regarding distribution of neutrophil, eosinophil or lymphocyte counts. Future functional studies of these cell populations over time, including investigation of cytokine release, may help to better understand this discrepancy and the likely underlying dynamic processes.

Similar to first-episode schizophrenia patients in our previous study [12], we found no significant difference in baseline lymphocyte counts in FEMD in the present report (Additional file 1: Table S7). However, there was a significant increase in lymphocyte counts in RMD, suggesting an involvement of the adaptive immune system. Consistent with our findings, a previous study reported increased lymphocyte counts in a longitudinal study of individuals with a current disease episode of MD [29] and two others showed that severity of depression and stress significantly interacted with increased lymphocyte counts in depressive patients [31, 34].

In both cohorts of patients, GAF and HAMD-21 scores decreased significantly following treatment with antidepressants, with stronger effects found in FEMD compared to RMD patients. Notably, we found that the decreased HAMD-21 symptom scores were paralleled by a decrease in neutrophil counts in FEMD but not in RMD. Thus, larger improvements in symptoms were associated with larger decreases in neutrophils in FEMD. This suggests that neutrophil counts have potential use as a biomarker of response to treatment in early-stage MD patients. However, as other physiological and pathological states can affect leucocyte count, the use of this as a biomarker of MD symptoms or treatment response may have low specificity. The medication-induced effect on larger treatment-related decreases in HAMD scores were associated with larger increases in eosinophils from T0 to T6 in RMD patients only. Furthermore, increased cumulative dose of amitriptyline were associated with reduced neutrophil counts in RMD patients only. This is in line with van Staa et al. [58] who observed an association of antidepressants with neutropenia and in rare cases, even with agranolocytosis. Kornhuber et al. [59] suggested that antidepressants may affect sphingomyelinase activity, which plays an important role in leukocyte homeostasis and function. The authors reported high activity of acid sphingomyelinase in non-medicated MD patients compared to controls and showed a counter-regulatory effect of imipramine and amitriptyline on PBMCs in culture.

We observed no association between CRP levels and symptom severity, which is in line with results from the large Netherlands Study of Depression and Anxiety (NESDA) [60]. Similar to schizophrenia patients, CRP levels were elevated in the present study in both FEMD and RMD patients with small effect sizes (Additional file 1: Table S7). We controlled for smoking which is relevant, because it is known that CRP levels are increased by smoking [61] and due to the higher percentage of smokers in FEMD and RMD compared to controls. Six-week treatment did not induce a significant change in CRP levels in either group, which is consistent with recent longitudinal meta-analyses [62, 63]. Taken together, these findings suggest that CRP may be a trait marker, while neutrophil and eosinophil counts are likely to be state markers of current depression.

The present study benefited from inclusion of clinically well-characterized FEMD and RMD patients and a longitudinal analysis to assess dynamic changes. Moreover, contrary to previous studies, potential confounders were considered (Table 2, inclusion of relevant covariates). Another strength is that this study focused on everyday routine blood parameters familiar to clinical psychiatrists, thus providing an easy comprehensible approach to the subject of psychoimmunology. However, some limitations should be considered. First, no functional or more precise cell subtype-specific characterization of neutrophils, eosinophils and lymphocytes were available. Second, cortisol measures were available only in a subgroup of 103 subjects. Conclusions about the role of cortisol, which was a secondary aim of this study, should therefore be interpreted with caution. In addition, the significance of altered patterns of neutrophil and eosinophil blood counts in current MD requires validation due to the present lack of data on microbial exposure in this cohort. In the future studies where the history of recent bacterial/viral infections is not available, we suggest that determination of the circulating levels of endotoxin, total IgG and neutrophil-released defensins should be considered. Finally, as MD is defined as a condition with depressed mood for at least 2 weeks and may also involve chronic dynamics, we note that it is difficult to compare patients with current episodes of this disease with acute schizophrenia.

## Conclusions

In summary, our findings suggest immunological differences regarding immune activation and inflammation status in relation to different stages of depression. A recent study showed that 165 immune-related genes are differentially expressed in MD patients compared to controls [64]. Additionally, multiple neutrophil and innate immunity-related genes were differentially expressed in depressed patients versus controls [65]. In the future, it will be important to gain more insight into the interplay between genetic vulnerability and environmental stressors or infectious disease-disposing factors. Apparently, neutrophil and eosinophil granulocytes as well as CRP have a more general role in major psychiatric disorders as these parameters were altered in both depression and schizophrenia. We identified raised neutrophil and reduced eosinophil counts in both FEMD and RMD patients with a current disease episode of MD. The effect sizes were smaller compared to schizophrenia, and antidepressants had a weaker association with decreased neutrophils during treatment as compared to the effects of antipsychotic treatment. In addition, different from schizophrenia, we found elevated lymphocytes at baseline in recurrent patients. These findings suggest chronic activation of the adaptive immune system during progression of MD. This may have implications for potential new treatment approaches which target different aspects of the immune system in a personalized medicine-based approach.

## Supplementary Information

**Additional file 1: Table S1.** Treatment status of patients. **Table S2.** Demographic, clinical and lab data of patients with *atypical MD* (Yes) versus *typical MD* patients (No). **Table S3.** Association of improvement in depressive symptoms (ΔHAMD-21 or ΔGAF) with changes in WBC and CRP from baseline to T6. **Table S4.** Demographic, clinical and lab data of *non-smoking* patients and controls. **Table S5.** Spearman correlation matrix between cortisol and WBC counts or clinical scores. *p*-values are FDR-corrected in each column. **Table S6.** Previous studies on WBC in patients with major depression. **Table S7.** Pattern of WBC counts and CRP alterations in major depression (present study) and schizophrenia (past study: Steiner et al. [12]).

## Data Availability

The data used and analyzed herein are available from the corresponding author upon reasonable request.

## Abbreviations

ANCOVA: Analysis of covariance
ART: Aligned Rank Transformation
BBB: Blood–brain barrier
CRP: C-reactive protein
DSM: Diagnostic and statistic manual of mental disorders
EDTA: Ethylenediaminetetraacetic acid
FEMD: First-episode of major depression
GAF: Global Assessment of Functioning
HAMD-21: 21-Item Hamilton depression scale
HPA: Hypothalamic–pituitary–adrenal
IFN-γ: Interferon-gamma
IL: Interleukin
LPS: Lipopolysaccharide
MD: Major depression
MINI: Mini-International Neuropsychiatric Interview
MIP-1α: Macrophage inflammatory protein-1 alpha
MLR: Monocyte-lymphocyte ratio
MPS: Mononuclear phagocyte system
NGAL: Neutrophil gelatinase associated lipocalin
NLR: Neutrophil–lymphocyte ratio
PBMCs: Peripheral blood mononuclear cells
RMD: Recurrent major depression
T0: Baseline
T6: After 6 weeks treatment
TNF-α: Tumor necrosis factor alpha
WBCs: White blood cell counts
