# Supplementary material for: Changes in leukocytes and CRP in different stages of major depression
Source: J Neuroinflammation. 2022 Apr 4;19:74. doi: 10.1186/s12974-022-02429-7 (PMC8981816; doi:10.1186/s12974-022-02429-7)
Supplement: Supplementary file 1 — Additional file 1: Table S1. Treatment status of patients. Table S2. Demographic, clinical and lab data of patients with atypical MD (Yes) versus typical MD patients (No). Table S3. Association of improvement in depressive symptoms (ΔHAMD-21 or ΔGAF) with changes in WBC and CRP from baseline to T6. Table S4. Demographic, clinical and lab data of non-smoking patients and controls. Table S5. Spearman correlation matrix between cortisol and WBC counts or clinical scores. p-values are FDR-corrected in each column. Table S6. Previous studies on WBC in patients with major depression. Table S7. Pattern of WBC counts and CRP alterations in major depression (present study) and schizophrenia (past study: Steiner et al. [12]). [file 12974_2022_2429_MOESM1_ESM.doc]

**Table S1.** Treatment status of patients.

| **Treatment** | **FEMD (number)** | **RMD (number)** |
| --- | --- | --- |
| **Agomelatine** | 2 | 2 |
| **NASSA** | 17 | 7 |
| **SNRI** | 19 | 13 |
| **SSRI** | 14 | 9 |
| **TCA** | 1 | 0 |
| **Psychotherapy** | 10 | 6 |

NASSA: Noradrenergic and specific serotonergic antidepressant

SNRI: Serotonin-noradrenalin reuptake inhibitor

SSRI: Selective serotonin reuptake inhibitor

TCA: tricyclic antidepressant

FEMD: first episode major depression

RMD: Relapsed major depression

**Table S2.** Demographic, clinical and lab data of patients with *atypical MD* (Yes) versus *typical MD* patients (No).

*Annotations:* Data are presented as median (quartile 1; quartile 3; sample size); significant p-values highlighted in green; **p*<0.05, ***p*<0.01, ****p*<0.001.

**Table S3.** Association of improvement in depressive symptoms (ΔHAMD-21 or ΔGAF) with changes in WBC and CRP from baseline to T6.

| **variables** | **Spearman correlation**  **FDR-corrected** | **Δ HAMD in FEMD** | **Δ HAMD in RMD** | **Δ GAF in FEMD** | **Δ GAF in RMD** |
| --- | --- | --- | --- | --- | --- |
| **Δ Neutrophils [×10^9^/L]** | r | 0.364 | 0.116 | -0.199 | -0.232 |
|  | p | **0.024*** | 0.607 | 0.763 | 0.360 |
|  | n | 61 | 35 | 58 | 35 |
| **Δ Eosinophils [×10^9^/L]** | r | -0.119 | -0.460 | 0.098 | 0.406 |
|  | p | 0.680 | **0.043*** | 0.763 | 0.114 |
|  | n | 61 | 33 | 58 | 33 |
| **Δ Basophils [×10^9^/L]** | r | -0.065 | 0.138 | -0.041 | 0.143 |
|  | p | 0.680 | 0.607 | 0.763 | 0.574 |
|  | n | 61 | 33 | 58 | 33 |
| **Δ Monocytes [×10^9^/L]** | r | -0.223 | -0.065 | -0.042 | 0.055 |
|  | p | 0.286 | 0.716 | 0.763 | 0.759 |
|  | n | 57 | 34 | 54 | 34 |
| **Δ Lymphocytes [×10^9^/L]** | r | 0.061 | -0.137 | 0.042 | 0.242 |
|  | p | 0.680 | 0.607 | 0.763 | 0.360 |
|  | n | 61 | 35 | 58 | 35 |
| **Δ CRP [mg/L]** | r | 0.053 | -0.283 | 0.073 | 0.130 |
|  | p | 0.680 | 0.350 | 0.763 | 0.574 |
|  | n | 62 | 32 | 60 | 32 |

**Annotations: p-values are FDR-corrected in each column, highlighted in green; * p< 0.05.**

**Table S4.** Demographic, clinical and lab data of *non-smoking* patients and controls. *Annotations:* Data are presented as median (quartile 1; quartile 3; sample size); significant FDR-corrected *p*-values are highlighted in green; **p*<0.05, ***p*<0.01, ****p*<0.001.

**Table S5.** Spearman correlation matrix between cortisol and WBC counts or clinical scores. *p*-values were FDR-corrected in each column.

**Table S6.** Previous studies on WBC in patients with major depression.

*Abbreviations:* BDI (Beck's Depression Inventory), CRP (C-reactive protein), FEMD (first episode of major depression), HAMD (Hamilton depression scale), HC (healthy control), MD (major depression), MADRS (Montgomery–Åsberg depression rating scale), MD<HC (cell count decreased in MD patients compared to HC), MD>HC (cell count increased in MD patients compared to HC), NA (not applicable), NLR (neutrophil-lymphocyte ratio), NM (not mentioned in the study), NS (not significant), QD2A (self-report Questionnaire of Depression, second version, abridged), RMD (relapse of major depression), S (significant), SHAPS (Snaith–Hamilton pleasure scale).

| **Author** | **Study design** | **Participants (per diagnostic group)** | **Diagnostic criteria** | **Disease stage**  **at time of blood take** | **FEMD/RMD**  **Duration of disease** | **Severity assessment** | **Status of medication in patients** | | **Consideration of potential confounder**  **Smoking**  **Stress** | **Differences in peripheral blood count (MD vs HC)** |
| --- | --- | --- | --- | --- | --- | --- | --- | --- | --- | --- |
|  |  |  |  |  |  |  | **Before** | **After** |  |  |
|  | Cross-sectional study | HC=30; median age 43±12 years,  MD=30; median age 44±12 years (unipolar, n = 18; median age 45±13 years and bipolar, n = 12; median age 43±12 years) | DSM-III | Unclear | Unclear  Duration of disease: **NM** | HAMD, BDI | 20 patients were free from medication ≥1 month and 10 patients received psychotropic medications immediately before  admission to the study, including tricyclic antidepressants (2), lithium carbonate (3), neuroleptics (2), lithium plus tricyclic (I), lithium plus neuroleptic (I), tricyclic plus alprazolam (l) | NA (cross-sectional study) | No (smoking not mentioned in the demographic data, and not considered for statistics)  Stress mentioned and divided into 3 groups on the basis of DSM-III Axis IV rating: Low, moderate and high  **no stress-associated WBC differences** | **MD vs HC**:  Baseline  **S**: MD>HC: Leukocytes and neutrophil counts.  MD<HC in % lymphocyte  **NS**: Eosinophil, monocyte and lymphocyte in counts.  Stress showed NS effect on WBC counts, however lymphocyte % rose along with increase in stress.  **NM**: basophil counts and CRP level in MD  **Severity correlation**:  **NS**: HAMD and BDI had no correlation with leukocytes, neutrophils, monocytes, eosinophils and lymphocytes in MD patients.  **NM:** Basophil and CRP level |
|  | Cross-sectional study | HC =36 non patients; mean age 42.1 ± 9.8; range 26-63 years), MD=36 in-patients; mean age (42.3± 10.6; range 24-67 years) | Research Diagnostic Criteria | Unclear | Unclear  Duration of disease: **NM** | HAMD | 25 patients were free from antidepressants > 3 months, 9 had undergone drug washout period and drug-free for 14 days to 30 days before testing. | NA (cross-sectional study) | Smoking correlated with leukocytes (r=+0.67, p<0.01), neutrophil (r=+0.67, p<0.01) and lymphocyte counts (r= + 0.41, p<0.01).  Stress was divided into two categories: low and high.  Depression and stress interacted significantly only for lymphocyte counts, **Interaction between Stress and depression showed no association with severity of depressive symptoms.** | **MD vs HC**:  Baseline  Stress:  **S**: Lymphocyte counts; In case of high stress lymphocyte count was high in MD.  **NS**: Leukocyte, neutrophil and monocyte counts Eosinophil, monocyte.  **NM**: Eosinophil, basophil counts and CRP level in MD  **Severity correlation**:  **S**: HAMD had correlation with leukocytes, neutrophil counts of MD  **NS**: Monocytes  **NM:** Basophils, eosinophils and CRP level |
|  | Cross-sectional study | HC=22;  mean age 42.4 ±2.8 years)  MD=79; mean age 45 ±1.8 years) | DSM-III | Unclear | Unclear  Duration of disease: **NM** | HAMD,  Severity of illness index range (0-3) | 50%: no drug intake >1 month. 50% of patients: washout period for 6-8 days | NA (cross-sectional study) | No (smoking and stress not mentioned in demographic data and not considered as confounding factor) | **MD vs HC**:  Baseline  **S**: MD>HC: Leukocytes, monocytes and neutrophils  **NS**: Eosinophils and basophils; ns increase of lymphocytes in MD  **NM**: CRP  **Severity correlation**:  **S**: Overall severity correlated with leukocytes, monocytes, neutrophils.  **NS**: Eosinophil, basophil and lymphocytes  **NM:** correlation with HAMD |
|  | Longitudinal study for six weeks. | HC =44; mean age 37.5 ±10.6, MD =33; mean age 39.8±9.0 | DSM-III-R | Current disease episode of depression | Unclear  Duration of disease: **NM** | HAMD | 50% patients were taking tricyclic antidepressants prior to the first examination but no difference in any tested parameters vs unmedicated patients. | Antidepressant treatment | No (smoking and stress not mentioned in demographic data and not considered as confounding factor) | Baseline  **S**: MD>HC: Leukocytes, monocytes, neutrophils and basophils  **NS**: Eosinophils and lymphocytes, Th, Treg, Tc,  **NM**: CRP, NLR  Follow-up to six weeks  **S**: T0>T6: Eosinophils, monocytes, lymphocytes  **NS**: Leukocytes, neutrophils, basophils, Th, Treg, Tc,  **Severity correlation**: NM |
|  | Cross-sectional study | HC = 47 (mean age: 30.0±9.2years)  MD= 41 (mean: 28.4±9.2years) | DSM-V | Unclear | Unclear  Duration of disease considered but not mentioned in demographic data of patients. | BDI | All patients did not receive antidepressant medication >1 month prior to hospital admission | NA (cross-sectional study) | Yes (smokers excluded from patient group)  Stress not mentioned in the demographic data and not considered for statistics. | **MD vs HC**:  Baseline  **S**: MD>HC; Leukocytes, neutrophils, NLR  **NS**: Lymphocyte count but significant increase in percentage  **NM**: Eosinophils and basophils, CRP  **Severity correlation**:  **S**: -  **NS**: BDI correlated with NLR of MD.  **NM:** BDI correlation with other blood parameters |
|  | Longitudinal study T0: at the time of diagnosis; T3: after 3 months of  treatment | HC= 91 (mean age: 39.80 ± 11.50 years), MD= 80 (mean age: 44.10 ± 10.60 years) | DSM-IV | Unclear | Unclear  Duration of disease: **NM** | MADRS | NM | SSRIs (Escitalopram and Sertraline) for 3 months.  Escitalopram: 10 to 20 mg/day and sertraline: 25 to 100 mg/day. | Smokers excluded from patient group hence not considered for statistics.  Stress was not mentioned in demographic data and not considered for statistics. | **MD vs HC**:  **S**: **Baseline;** MD>HC in NLR. **Follow-up**; T0>T3 in NLR of MD patients  **NM**: Leukocytes, monocytes, eosinophils, basophils and CRP  **Severity correlation**  **NM** |
|  | Cross-sectional study | HC =106 (mean age:46.58±13.38 years)  MD= 103 (mean ge:46.58±13.38 years) | DSM-IV | Current disease episode of depression (blood was taken as first day admission to the inpatients unit) | Unclear  Duration of disease: **NM** | HAMD | No antidepressant medication >one month prior to hospital admission | NA (cross-sectional study) | No (Smoking and stress did not mention in demographic data and not consider as confounding factor.) | **MD vs HC**:  Baseline  **S**: MD>HC; Leukocytes, neutrophil and NLR.  MD<HC; Lymphocytes.  **NM**: Eosinophil, monocytes, basophil and CRP  **Severity correlation**: NM |
|  | Cross-sectional study | HC = 30 (mean age: 37.3 ±12.2 years), MD= 98 (mean age: 37.3±10.8 years) | DSM-IV | Unclear  (outpatients) | Unclear  Duration of disease: **NM** | BDI-II | Patients who took antidepressants were considered for participation under the assumption that the dose had been stable for at least 2 weeks and would remain so during study participation | NA (cross-sectional study) | Smoking mentioned in the demographic data but considered statistics only for CRP and NLR.  Stress not mentioned in the demographic data, stress-related parameters not considered for statistics) | **MD vs HC**:  Baseline  **S**: MD>HC; Neutrophil, monocytes, CRP and trend was found Treg.  **CF:** Age and sex; still CRP NLR were high in MD  Smoking: still CRP high, hence smoking significantly effect on NLR of MD  **NS**: Leukocytes, lymphocytes  **NM**: Eosinophil and basophils  **Severity correlation**:  **NM** |
|  | Cross-sectional study | HC = 121; mean age 14.46 ±1.77 years, MD= 67 adolescent patients; mean age 14.47 ±1.85 years (12-18 years) | DSM-V | Unclear (outpatients) | 8.98±3.4 weeks duration of disease but not clear whether FEMD or RMD | BDI | 67 drug-naïve depressive adolescents aged 12-18 years with no  psychiatric comorbidities were enrolled in patient group | NA (cross-sectional study) | No (smokers of more than 15 cigarettes per day were excluded, but smoking was not mentioned in the demographic data. Smoking not considered for statistics).  No (stress was not mentioned in the demographic data, stress-related parameters were not considered for statistics) | **MD vs HC**:  Baseline adjusted with age and sex  **S**: MD>HC; Leukocytes and neutrophil counts, NLR  **NS**: Lymphocytes  **NM**: Eosinophils, basophils, monocytes and CRP  **Severity correlation**:  **S:** NLR correlated with BDI and duration of Illness.  **NM:** correlation of BDI and duration of illness with other counts. |
|  | Retrospective, cross-sectional study | Non-depressed= 219; mean age: 71.1 ± 5.7 years). MD= 465; mean age 74.8 ± 7.8 years) | ICD-10 criteria, F32 (first episode)/F33 (recurrent  depression) | Current disease episode of depression | FEMD: n=138, 29.6% and RMD: n=328, 70.3% | Severity of depression (mild, moderate, severe  or severe with psychotic symptoms) was based on ICD-10  codes | FEMD: 45.6% unmedicated and 55.4 % medicated. RMD: 32.1 % unmedicated, 67.9 medicated (antidepressants, antipsychotics, mood stabilizers and benzodiazepines). No differences for the use of various antidepressants between FEMD and RMD patients. | NA (cross-sectional study) | No (smoking was not mentioned in the demographic data, smoking not considered for statistics)  No (stress was not mentioned in the demographic data, stress-related parameters not considered for statistics) | **MD vs HC**:  Baseline adjusted with sex  **S**: NLR, MD>HC but after using post-hoc Dunn's pairwise comparison FEMD>HC; p<0.001, FEMD>RMD p=0.05  **NM**: Leukocytes, neutrophils, eosinophils, basophils, monocytes, lymphocytes and CRP  **Severity correlation**:  **S:** NLR related to ICD-10 severity  **NM:** correlation of BDI and duration of illness with other counts.  No correlation between NLR and age  No effect of antidepressant drugs on NLR |
|  | Case-control study | HC = 77(mean age:32.5 (28.3, 39.1)), MD= 206 (mean age: 35.3 (28.7, 42.9)) | DSM screening questionnaire | Unclear | Unclear  Duration of disease: **NM** | HAMD, BDI-II, Chalder Fatigue Scale, SHAPS | Patients who took antidepressants were considered for participation under the assumption that the dose had been stable for at least 2 weeks and would remain unchanged during study participation. | NA (cross-sectional study) | No (smoking and stress mentioned in demographic data, but not considered as confounding factor). | **MD vs HC**:  Baseline  **S**: MD>HC; Neutrophils, monocytes, T cells and CRP  **NS**: Eosinophils, basophils  **NM**: Lymphocytes and NLR  **Severity correlation**:  **S:** BDI and HAMD correlated with neutrophil counts in MD.  **NS**: Monocytes, T cells and CRP. |

**References:**

Arabska, J., Lucka, A., Magierski, R., Sobow, T., Wysokinski, A., 2018. Neutrophil-lymphocyte ratio is increased in elderly patients with first episode depression, but not in recurrent depression. Psychiatry Res 263, 35-40.

Cai, L., Xu, L., Wei, L., Chen, W., 2017. Relationship of Mean Platelet Volume To MDD: A Retrospective Study. Shanghai archives of psychiatry 29, 21-29.

Darko, D.F., Rose, J., Gillin, J.C., Golshan, S., Baird, S.M., 1988. Neutrophilia and lymphopenia in major mood disorders. Psychiatry Res 25, 243-251.

Demir, S., Atli, A., Bulut, M., Ibiloglu, A.O., Gunes, M., Kaya, M.C., Demirpence, O., Sir, A., 2015. Neutrophil-lymphocyte ratio in patients with major depressive disorder undergoing no pharmacological therapy. Neuropsychiatric disease and treatment 11, 2253-2258.

Demircan, F., Gozel, N., Kilinc, F., Ulu, R., Atmaca, M., 2016. The Impact of Red Blood Cell Distribution Width and Neutrophil/Lymphocyte Ratio on the Diagnosis of Major Depressive Disorder. Neurology and therapy 5, 27-33.

Euteneuer, F., Dannehl, K., Del Rey, A., Engler, H., Schedlowski, M., Rief, W., 2017. Peripheral Immune Alterations in Major Depression: The Role of Subtypes and Pathogenetic Characteristics. Frontiers in psychiatry 8, 250.

Irwin, M., Patterson, T., Smith, T.L., Caldwell, C., Brown, S.A., Gillin, J.C., Grant, I., 1990. Reduction of immune function in life stress and depression. Biol Psychiatry 27, 22-30.

Lynall, M.E., Turner, L., Bhatti, J., Cavanagh, J., de Boer, P., Mondelli, V., Jones, D., Drevets, W.C., Cowen, P., Harrison, N.A., Pariante, C.M., Pointon, L., Clatworthy, M.R., Bullmore, E., Neuroimmunology of Mood, D., Alzheimer's Disease, C., 2020. Peripheral Blood Cell-Stratified Subgroups of Inflamed Depression. Biol Psychiatry 88, 185-196.

Maes, M., Van der Planken, M., Stevens, W.J., Peeters, D., DeClerck, L.S., Bridts, C.H., Schotte, C., Cosyns, P., 1992. Leukocytosis, monocytosis and neutrophilia: hallmarks of severe depression. J Psychiatr Res 26, 125-134.

Özyurt, G., Binici, N.C., 2018. Increased neutrophil-lymphocyte ratios in depressive adolescents is correlated with the severity of depression. Psychiatry Res 268, 426-431.

Seidel, A., Arolt, V., Hunstiger, M., Rink, L., Behnisch, A., Kirchner, H., 1996. Major depressive disorder is associated with elevated monocyte counts. Acta Psychiatr Scand 94, 198-204.

**Table S7.** Pattern of WBC counts and CRP alterations in **major depression** (present study) and **schizophrenia** .

**a)** To compare these studies, WBC counts and CRP in major depression (FEMD, RMD) vs. controls were analyzed using **H-tests** and **U-tests,** as in the previous schizophrenia study.

*Annotation:* the same data were calculated by ART in Table 3 of the main manuscript. Data are presented as median (quartile 1; quartile 3; sample size). Significant p-values highlighted in green; * x10^9^/L.

| **variables** | **FEMD** | **RMD** | **Control** | **test value** | **p-value** | ***test*** | ***p post-hoc U-test.FEMD-C FDR*** | ***Cliff's delta.FEMD-C*** | ***p post-hoc U-test.RMD-C FDR*** | ***Cliff's delta.RMD-C*** | ***p post-hoc U-test.FEMD-RMD FDR*** | ***Cliff's delta.FEMD-RMD*** |
| --- | --- | --- | --- | --- | --- | --- | --- | --- | --- | --- | --- | --- |
| Neutrophils-T0 * | 4.22 (3.43;5.37;81) | 4.52 (3.42;5.39;46) | 3.37 (2.66;4.40;128) | KW=23.51 | **<0.001** | H-test | **<0.001***** | 0.329 | **<0.001***** | 0.373 | 0.557 | -0.050 |
| Neutrophils-T6 * | 3.79 (3.02;5.06;62) | 3.87 (3.12;5.31;35) | - | W=1093.5 | 0.952 | U-test |  |  |  |  |  | -0.013 |
| Test value | W=1128 | W=419 |  |  |  |  |  |  |  |  |  |  |
| p-value | 0.067 | 0.090 |  |  |  |  |  |  |  |  |  |  |
|  |  |  |  |  |  |  |  |  |  |  |  |  |
| Eosinophils-T0 * | 0.14 (0.07;0.20;81) | 0.14 (0.07;0.20;44) | 0.15 (0.10;0.26;128) | KW=5.71 | 0.058 | H-test | 0.100 | -0.166 | 0.100 | -0.211 | 0.796 | 0.051 |
| Eosinophils-T6 * | 0.20 (0.12;0.32;62) | 0.13 (0.08;0.20;34) | - | W=1299.0 | 0.061 | U-test |  |  |  |  |  | 0.223 |
| Test value | W=544.5 | W=125 |  |  |  |  |  |  |  |  |  |  |
| p-value | **0.010** | **0.028** |  |  |  |  |  |  |  |  |  |  |
|  |  |  |  |  |  |  |  |  |  |  |  |  |
| Basophils-T0 * | 0.05 (0.00;0.07;81) | 0 (0;0.07;44) | 0.05 (0;0.07;128) | KW=1.62 | 0.445 | H-test | 0.518 | 0.052 | 0.469 | -0.086 | 0.469 | 0.104 |
| Basophils-T6 * | 0.05 (0.00;0.07;62) | 0 (0;0.07;34) | - | W=1120.0 | 0.593 | U-test |  |  |  |  |  | 0.068 |
| Test value | W=611 | W=98.5 |  |  |  |  |  |  |  |  |  |  |
| p-value | 0.294 | 0.823 |  |  |  |  |  |  |  |  |  |  |
|  |  |  |  |  |  |  |  |  |  |  |  |  |
| Monocytes-T0 * | 0.45 (0.32;0.61;78) | 0.41 (0.32;0.56;44) | 0.40 (0.32;0.50;128) | KW = 4.29 | 0.117 | H-test | 0.128 | 0.169 | 0.481 | 0.080 | 0.515 | 0.093 |
| Monocytes-T6 * | 0.43 (0.34;0.54;60) | 0.45 (0.33;0.58;35) | - | W = 994.0 | 0.669 | U-test |  |  |  |  |  | -0.079 |
| Test value | W=1029.5 | W=244 |  |  |  |  |  |  |  |  |  |  |
| p-value | 0.108 | 0.370 |  |  |  |  |  |  |  |  |  |  |
|  |  |  |  |  |  |  |  |  |  |  |  |  |
| Lymphocytes-T0 * | 2.08 (1.66;2.52;81) | 2.48 (1.93;2.96;46) | 1.90 (1.52;2.38;128) | KW=16.67 | **<0.001** | H-test | 0.081 | 0.144 | **<0.001***** | 0.386 | **0.012*** | -0.278 |
| Lymphocytes-T6 * | 2.27 (1.77;2.69;62) | 2.24 (1.92;2.55;35) | - | W=1080.0 | 0.973 | U-test |  |  |  |  |  | -0.006 |
| Test value | W=663 | W=389 |  |  |  |  |  |  |  |  |  |  |
| p-value | **0.043** | 0.232 |  |  |  |  |  |  |  |  |  |  |
|  |  |  |  |  |  |  |  |  |  |  |  |  |
| CRP-T0 (mg//L) | 3.61 (1.00;4.00;82) | 2.00 (1.00;4.00;45) | 1 (1;3;129) | KW=17.63 | **<0.001** | H-test | **<0.001***** | 0.317 | **0.009**** | 0.259 | 0.775 | 0.053 |
| CRP-T6 (mg(L) | 1.75 (0.70;4.00;62) | 1.90 (0.80;4.00;32) | - | W=946.0 | 0.716 | U-test |  |  |  |  |  | -0.063 |
| Test value | W=745 | W=187.5 |  |  |  |  |  |  |  |  |  |  |
| p-value | 0.797 | 0.981 |  |  |  |  |  |  |  |  |  |  |

**b)** As a next step, **Effect sizes** of both studies were compared by **Cliff's delta** (ǀδǀ≥0.147 “small”, ǀδǀ≥0.330 “medium”, ǀδǀ≥0.474 “large” effect size):

|  | **C (n=129/294)** | **FEMD (n=82)** | **RMD (n=47)** | **FEP (n=129)** | **Sz (n=124)** |
| --- | --- | --- | --- | --- | --- |
|  |  | **Major Depression** | | **Schizophrenia** | |
| **Neutrophils** | T0 vs. C | 🡹 0.329 (small) | 🡹🡹 0.373 (medium) | 🡹🡹🡹 0.529 (large) | 🡹🡹🡹 0.508 (large) |
|  | Follow-up vs. T0 | ↓ only trend | ↓ only trend | **↓** | **↓** |
| **Eosinophils** | T0 vs. C | ↓ only trend | ↓ only trend | **🡻** 0.312 (small) | **🡻** 0.168 (small) |
|  | Follow-up vs. T0 | **↑** | **↓** | **↑** | **↑** |
| **Basophils** | T0 vs. C | ↔ | ↔ | ↔ | ↔ |
|  | Follow-up vs. T0 | ↔ | ↔ | ↔ | ↔ |
| **Monocytes** | T0 vs. C | ↔ | ↔ | 🡹 0.262 (small) | 🡹🡹 0.385 (medium) |
|  | Follow-up vs. T0 | ↔ | ↔ | ↔ | **↓** |
| **Lymphocytes** | T0 vs. C | ↑ only trend | 🡹🡹 0.386 (medium) | ↔ | ↔ |
|  | Follow-up vs. T0 | **↑** | ↔ | ↔ | ↔ |
| **CRP** | T0 vs. C | 🡹 0.317 (small) | 🡹 0.259 (small) | 🡹 0.214 (small) | 🡹🡹 0.436 (medium) |
|  | Follow-up vs. T0 | ↔ | ↔ | ↔ | **↓** |
